# Clinical significance of precedent asymptomatic non-sustained ventricular tachycardias on subsequent ICD interventions and heart failure hospitalization in primary prevention ICD patients

**DOI:** 10.1186/s40001-020-0401-x

**Published:** 2020-03-17

**Authors:** Hisaki Makimoto, Sophie Zielke, Lukas Clasen, Tina Lin, Shqipe Gerguri, Patrick Müller, Jan Schmidt, Alexandru Bejinariu, Muhammed Kurt, Christoph Brinkmeyer, Manuel Stern, Malte Kelm, Alexander Fürnkranz

**Affiliations:** 1 Division of Cardiology, Pulmonology and Vascular Medicine, Medical Faculty, Heinrich-Heine-University, Moorenstrasse 5, 40225 Düsseldorf, Germany; 2 CARID, Cardiovascular Research Institute Düsseldorf, Medical Faculty, Heinrich-Heine-University, Moorensstrasse 5, 40225 Düsseldorf, Germany; 3 GenesisCare, East Melbourne, VIC Australia

**Keywords:** Non-sustained ventricular tachycardia, Implantable cardioverter-defibrillator, Ischemic cardiomyopathy, Non-ischemic cardiomyopathy

## Abstract

**Background:**

The prognostic implications of non-sustained ventricular tachycardia (NSVT) and their significance as therapeutic targets in patients without prior sustained ventricular arrhythmias remain undetermined. The aim of this study was to investigate the prognostic significance of asymptomatic NSVT in patients who had primary prevention implantable cardioverter-defibrillator (ICD) implantation due to ischemic or non-ischemic cardiomyopathy (ICM, NICM).

**Methods:**

We enrolled 157 consecutive primary prevention ICD patients without previous appropriate ICD therapy (AIT). Patients were allocated to two groups depending on the presence or absence of NSVT in a 6-month period prior to enrollment. The incidence of AIT and unplanned hospitalization due to decompensated heart failure (HF) were assessed during follow-up.

**Results:**

In 51 patients (32%), precedent NSVT was documented. During a median follow-up of 1011 days, AIT occurred in 36 patients (23%) and unplanned HF hospitalization was observed in 32 patients (20%). In precedent NSVT patients, the incidence of AIT and unplanned HF hospitalization was significantly higher as compared to patients without precedent NSVT (AIT: 29/51 [57%] vs. 7/106 [7%], *P* < 0.001, log-rank; HF hospitalization: 16/51 [31%] vs. 16/106 [15%], *P* = 0.043, log-rank). Cox-regression demonstrated that precedent NSVT independently predicted AIT (*P* < 0.0001). In subgroup analyses, precedent NSVT predicted AIT in both ICM and NICM (*P* < 0.0001, *P* = 0.020), but predicted HF hospitalization only in patients with ICM (*P* = 0.0030).

**Conclusions:**

Precedent non-sustained VT in patients with primary prevention ICDs is associated with subsequent appropriate ICD therapies, and is an independent predictor of unplanned heart failure hospitalizations in patients with ischemic cardiomyopathy.

## Background

Sudden cardiac death is still a leading cause of death worldwide in patients with structural heart disease. Multiple clinical trials have demonstrated that implantable cardioverter-defibrillator (ICD) therapy reduces sudden arrhythmic death due to ventricular tachycardia (VT) and ventricular fibrillation (VF) in patients with left ventricular dysfunction and improves patients’ prognosis [1–4].

Although ICD therapy improves patient survival, ICDs do not prevent the occurrence of VT/VF themselves. Defibrillation via ICD may result in considerable pain and often leads to a significant reduction in quality-of-life. Moreover, higher incidences of appropriate (and inappropriate) ICD therapy (AIT) and anti-tachycardia pacing (ATP) are reported to be associated with a poorer prognosis [5, 6]. These observations underline the importance of adequate rhythm control to suppress the incidence of ventricular tachyarrhythmias.

According to the current guidelines, rhythm control therapies such as amiodarone or catheter ablation are reserved for secondary prevention after sustained VT/VF or ICD shocks, or for symptomatic premature ventricular complexes (PVC)/non-sustained VT (NSVT) [3, 4]. Despite multiple studies which have demonstrated the potential aggravating effect of NSVT, there is still uncertainty on whether early proactive rhythm control strategies targeting asymptomatic NSVT without a prior history of sustained ventricular tachyarrhythmias are beneficial for patients with structural heart disease, who are already treated with optimal medical therapy [7–12].

We hypothesized that asymptomatic NSVT predicts the occurrence of subsequent ventricular tachyarrhythmias triggering AIT in patients with structural heart disease after primary prevention ICD implantation. This may have implications for early anti-arrhythmic therapy in this common patient population.

## Methods

### Study population

We consecutively enrolled patients who presented to our device clinics for routine follow-up between January and March 2015, who had undergone primary prophylactic ICD implantation according to current guidelines at least 6 months prior to the index presentation [3, 4]. This 6-month period was defined to ensure that a period of assessment can be performed for the presence or absence of NSVT in the ICD recordings.

Patients were excluded if they had undergone ICD implantation within the prior 6 months, if device interrogation had documented previous sustained VT/VF or AIT, or if they had experienced symptomatic PVCs or NSVT. The patients were also excluded if the implanted ICD was a subcutaneous ICD due to the different ICD settings. The patients who had worsening heart failure or myocardial infarction within the prior 6 months were excluded. Patients treated with amiodarone or sotalol were also excluded.

This study was conducted in the context of the Düsseldorf University Device Registry and approved by the local institutional review board. All patients gave written informed consent.

### Device programming and interrogation

All devices were programmed as per protocol according to the institutional standard [13]. The monitor zone without therapy was programmed at ≥ 150 beats per minute (bpm). A VT zone was programmed from 180 to 210 bpm, with the detection counter set to 38 (redetection counter 20). The onset and stability criteria were according to the manufacturer’s default settings. The programmed interventions were 4 × 8 beat bursts of anti-tachycardia pacing (ATP) at 88% coupling of the cycle length, followed by 4 × 8 beat ramps at 88% coupling of the cycle length with a 10-ms decrement. As the second therapy, the maximum number of biphasic shocks of 40 J (maximum shock energy) was programmed. A VF zone was set at > 210 bpm, with the detection counter set to 18 out of 24 intervals, and intervention was programmed to deliver biphasic shocks at 40 J. For Boston Scientific ICDs, VT/VF duration parameters were set at 12/5 s which is approximate to the device settings of the ICDs from other manufacturers. ATP in the VF zone was also programmed (Boston Scientific Quick Convert, Biotronik one-shot ATP, Medtronic ATP during charging, St. Jude Medical ATP Prior to Charging, ATP While Charging). If the precise setting was not available in a particular device, the nearest program setting was used.

### Patient allocation and data collection

The enrolled patients were allocated to two groups depending on the presence or absence of recorded NSVT within the 6 months prior to the index ICD interrogation. NSVT was defined as 3 or more successive ventricular beats at a rate over 150 beats/min and lasting < 30 s. Prospective patient follow-up started after the index ICD check was completed. Patients were followed up routinely in our device clinic. Follow-up data, including clinical parameters, device parameters and the occurrence of ICD therapies, were collected in a prospective database.

### Adjudication of events and therapy

Appropriate therapy was defined as ATP or shock rendered for VT or VF. Inappropriate therapy was defined as ATP or shock rendered when VT or VF was not present.

All incidents of ICD therapy were judged based upon the intracardiac electrogram. To adjudicate the arrhythmias, two independent electrophysiologists categorized the underlying cause/rhythm present at the time of therapy. If the two electrophysiologists did not reach an agreement, an additional electrophysiologist participated in the arrhythmia assessment.

### Clinical endpoints

The primary endpoint of this study was the occurrence of sustained (> 30 s) VT/VF or AIT during follow-up. Rhythm documentation of VT/VF was obtained by the way of ICD storage or any means of ECG. Secondary endpoints were the occurrence of unplanned hospitalization due to worsening heart failure (HF) and all-cause mortality. Unplanned hospitalization due to worsening HF was defined as an unplanned overnight hospital admission due to worsening of subjective/objective signs of congestive HF and the necessity of adjunctive therapies including an increase of oral diuretics, intravenous administration of diuretics, vasodilator and inotropic agents. The signs of congestive HF included pulmonary rales, worsening dyspnea, peripheral edema, increased N-terminal pro-BNP above baseline or radiological evidence of pulmonary congestion.

### Statistical analysis

Continuous data were shown as mean ± SD for normally distributed data. In cases of non-normal distributed data, these were shown as median values (lower–upper quartile). Categorical data were shown as numbers and percentages. The chi-square test, Kruskal–Wallis test, Student *t* test, Fisher’s exact test, or 1-way analysis of variance was performed when appropriate. Time to events was analyzed according to the Kaplan–Meier method and was compared using the log-rank test. To evaluate the association of clinical baseline variables with the primary or secondary endpoints, Cox regression analysis was performed. After verifying that the proportional hazard assumption was satisfied, multivariable Cox regression analysis was conducted incorporating all variables with a *P* value < 0.1 in the univariable analysis. For global test statistics, we used a significance level of 5%. Analyses were performed using JMP (SAS, Version 11) and EZR (Saitama Medical Center, Jichi Medical University, Saitama, Japan).

## Results

### Patients and devices

A total of 157 consecutive patients were enrolled. Baseline patient characteristics are shown in Table 1. The manufacturers of implanted ICDs included Boston Scientific in 87 cases (55%), Biotronik in 57 cases (36%), Medtronic in 12 cases (8%), and St. Jude Medical in 1 case. Seventy-six patients (48%) had a single-chamber ICD, 10 patients (6%) had a single-chamber ICD with an atrial sensing-electrode, 21 patients (13%) had a dual-chamber ICD, and 50 patients (32%) had a CRT-D.

**Table 1:** Baseline patients’ characteristics

	Total (*N* = 157)	NSVT (+) (*N* = 51)	NSVT (−) (*N* = 106)	*P* value
Age (years)	66.0 ± 12.1	65.7 ± 11.4	66.2 ± 12.5	0.80
Male	135 (86%)	45 (88%)	90 (85%)	0.57
NYHA				
Class I	21 (13%)	8 (16%)	13 (12%)	0.62
Class II	105 (67%)	35 (68%)	70 (66%)	
Class III	31 (20%)	8 (16%)	23 (22%)	
VVI-ICD	76 (48%)	24 (47%)	52 (49%)	0.79
VDD/DDD-ICD	31 (20%)	9 (18%)	22 (21%)	
CRT-D	50 (32%)	18 (35%)	32 (30%)	
Hypertension	113 (72%)	39 (76%)	74 (70%)	0.38
Diabetes mellitus	48 (31%)	18 (35%)	30 (28%)	0.37
Serum creatinine	1.29 ± 0.78	1.16 ± 0.31	1.35 ± 0.92	0.17
Hemoglobin (g/dL)	13.8 ± 1.8	14.0 ± 1.6	13.8 ± 1.8	0.53
Atrial fibrillation	60 (38%)	16 (31%)	44 (42%)	0.22
Ischemic cardiomyopathy	96 (61%)	38 (75%)	58 (55%)	0.017
LVEF (%)	31.5 ± 9.1	32.5 ± 10.4	31.1 ± 8.4	0.38
β Blocker	150 (96%)	49 (96%)	101 (95%)	0.82
ACEi or ARB	146 (93%)	47 (92%)	99 (93%)	0.78

*ACEi* angiotensin-converting-enzyme inhibitor, *ARB* angiotensin II receptor blocker, *LVEF* left ventricular ejection fraction, *NYHA* New York Heart Association

### Precedent NSVT

Out of the 157 enrolled patients, precedent NSVT was documented during the index ICD check in 51 patients (32%). All NSVT events were documented by ICD interrogation. The median number of documented NSVT within the prior 6 months in the patients with documented NSVT was 3.0 (q1–q3; 1.0–8.0), and the median duration of NSVT was 6.0 s (q1–q3; 2.8–10) and 12 beats (q1–q3; 8–23).

There were no significant differences in clinical baseline characteristics between patients with and without precedent NSVT, except for the prevalence of ischemic cardiomyopathy (Table 1) which was more prevalent in the patients with precedent NSVT (38/51 [75%] vs. 58/106 [55%], *P* = 0.017).

There was no significant difference in the LVEF between the groups (32.5 ± 10.4% vs 31.1 ± 8.4%, *P* = 0.38). The use of β-blocker and angiotensin-converting enzyme inhibitor (ACEi) or angiotensin-receptor blocker (ARB) was over 90% in both groups and did not differ significantly.

The median follow-up period was 1011 days (q1–q3; 905–1092), and there was no significant difference in the follow-up period between the two groups (1032 days [903–1127] in patients with NSVT vs 1011 days [909–1073] in patients without NSVT, *P* = 0.21).

### Primary endpoint

During the follow-up period, appropriate ICD therapy was observed in 36 patients (23%). There were no sustained VT events over 30 s without AIT in the monitor zone. As shown in Table 2, out of these 36 patients, 9 patients (25%) experienced both ATP and shock therapy. Two patients experienced only shock therapy due to VF and the remaining 25 patients only received ATP therapy.

**Table 2:** Observed primary endpoints

	Total *N *= 157	NSVT (+) *N* = 51	NSVT (−) *N* = 106
AIT	36 (23%)	29 (57%)	7 (7%)
Shock	11 (7%)	8 (16%)	3 (3%)
ATP	34 (22%)	28 (55%)	6 (6%)
Both	9 (6%)	7 (14%)	2 (2%)

*AIT* appropriate ICD therapy, *ATP* anti-tachycardia pacing

**Fig. 1:**
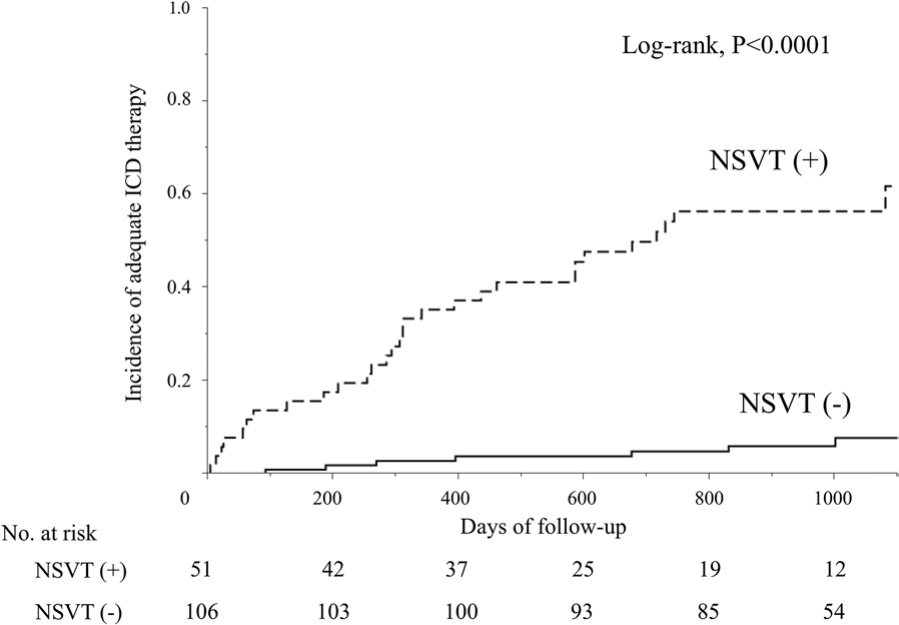
Kaplan–Meier analysis of appropriate ICD therapy according to the presence of precedent non-sustained ventricular tachycardia. Precedent non-sustained ventricular tachycardia (NSVT) predicted subsequent occurrences of appropriate ICD therapy (log-rank, *P* < 0.0001)

Overall, patients with precedent NSVT had a higher incidence of AIT compared to those without NSVT (29/51 [57%] vs. 7/106 [7%], log-rank, *P* < 0.0001, Fig. 1).

**Fig. 2:**
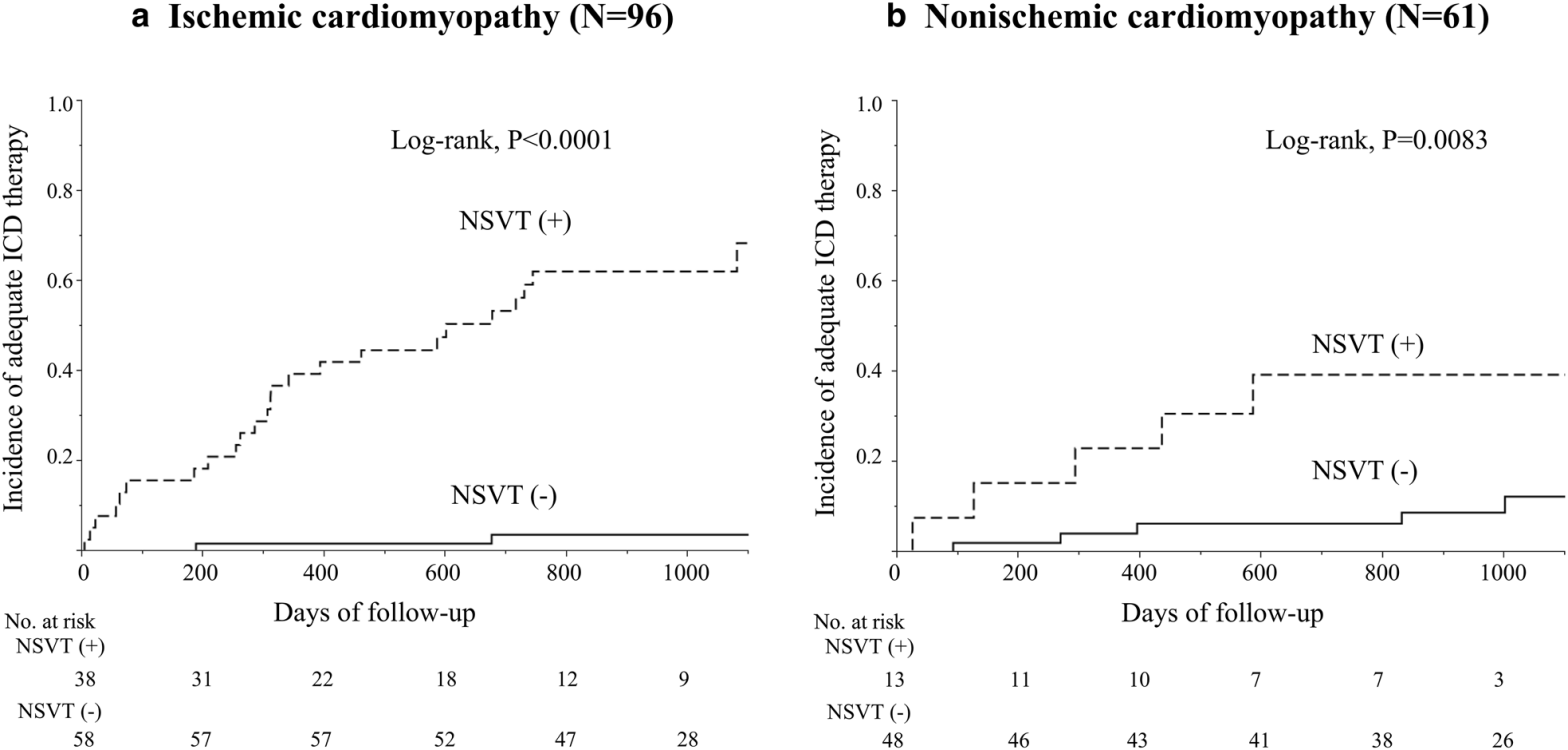
Subgroup Kaplan–Meier analyses of appropriate ICD therapy according to the presence of precedent non-sustained ventricular tachycardia. In both patients with ischemic (**a**) and non-ischemic cardiomyopathy (**b**), precedent non-sustained ventricular tachycardia was associated with subsequent appropriate ICD therapy (log-rank, *P* < 0.0001, and *P* = 0.0083, respectively)

The higher incidence of AIT in the NSVT group was demonstrated in both subgroups of ICM (*N* = 96, log-rank, *P* < 0.0001, Fig. 2a) and NICM patients (*N* = 61, log-rank, *P* = 0.0083, Fig. 2b).

There were no significant differences in the incidence of AIT between the types of ICDs (VVI-ICD 21/76 [28%], VDD/DDD-ICD 7/31 [23%], or CRT-D 8/50 [16%], *P* = 0.31).

### Secondary endpoints

**Fig. 3:**
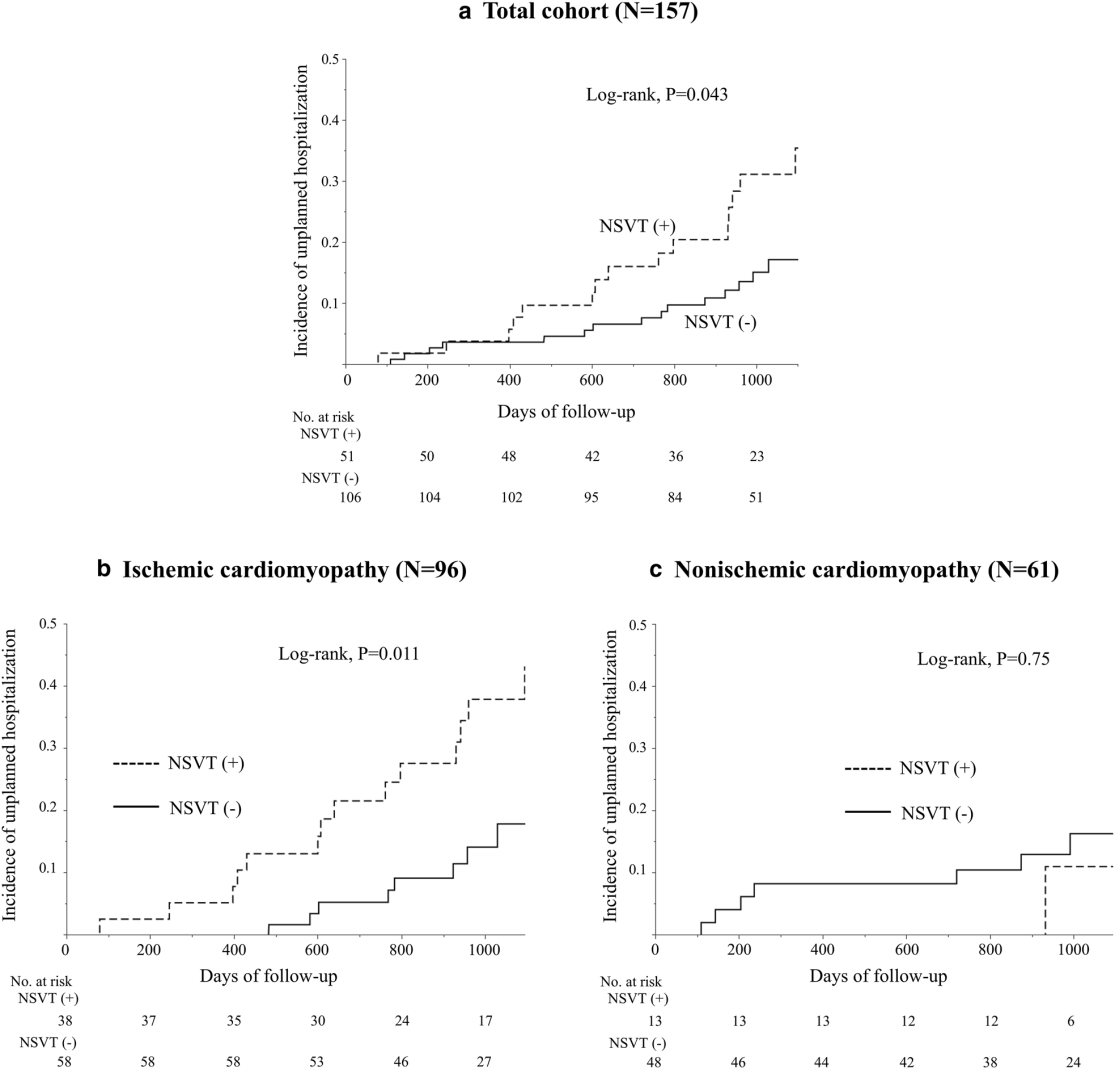
Kaplan–Meier analysis of unplanned hospitalization due to worsening heart failure according to the presence of precedent non-sustained ventricular tachycardia. Precedent non-sustained ventricular tachycardia (NSVT) predicted unplanned hospitalizations due to heart failure (HF) during follow-up (**a** log-rank, *P* = 0.043). In subgroup analyses, precedent NSVT was associated with higher unplanned HF hospitalization rates in patients with ischemic cardiomyopathy (**b** log-rank, *P* = 0.011), but not in patients with non-ischemic cardiomyopathy (**c** log-rank, *P* = 0.75)

During the follow-up period, unplanned hospitalization due to worsening HF was noted in 32 patients (20%). The patients with precedent NSVT had a higher incidence of hospitalizations due to worsening HF as compared to those without NSVT (16/51 [31%] vs. 16/106 [15%], log-rank, *P* = 0.043, Fig. 3a).

In the subgroup of ICM patients, precedent NSVT predicted unplanned hospitalizations during follow-up (log-rank, *P* = 0.011, Fig. 3b). In NICM patients, precedent NSVT was not associated with an increased incidence of unplanned hospitalizations (log-rank, *P* = 0.75, Fig. 3c).

**Fig. 4:**
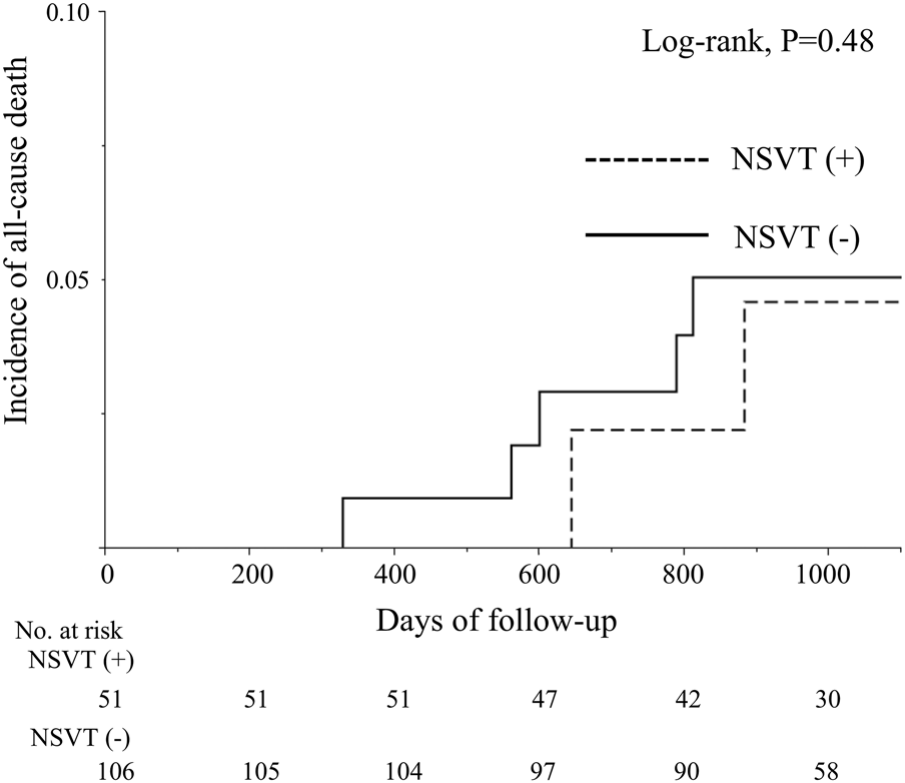
Kaplan–Meier analysis of all-cause mortality according to the presence of precedent non-sustained ventricular tachycardia. There was no significant difference in all-cause mortality between patients with and without precedent non-sustained ventricular tachycardia (*P* = 0.48)

Eight patients (5%) died during follow-up. There was no significant difference in the all-cause mortality between the two groups (log-rank, *P* = 0.48, Fig. 4).

### Predictors of outcome

In regard to the primary endpoint, in univariable analyses precedent NSVT was a significant predictor of subsequent AIT during follow-up (hazard ratio [HR]: 12.1, 95% confidence interval [CI] 5.29–27.7, *P* < 0.0001). Precedent NSVT remained a significant predictor of subsequent AIT after adjustments for age, sex, ischemic etiology and LVEF (HR: 11.8, 95% CI 5.10–27.3, *P* < 0.0001). As shown in Table 3, precedent NSVT was an independent predictor of subsequent AIT in both ICM and NICM patients.

**Table 3:** Predictors of appropriate ICD therapy

	Ischemic cardiomyopathy				Non-ischemic cardiomyopathy			
	Univariable analysis		Adjusted model^a^		Univariable analysis		Adjusted model^a^	
	Hazard ratio	*P* value	Hazard ratio	*P* value	Hazard ratio	*P* value	Hazard ratio	*P* value
LVEF (+1%)	1.00 [0.96–1.04]	0.94	1.00 [0.95–1.04]	0.86	0.97 [0.90–1.05]	0.40	0.97 [0.90–1.04]	0.42
NSVT	26.9 [6.33–114]	< 0.0001	26.1 [6.13–111]	< 0.0001	4.62 [1.33–16.1]	0.016	4.41 [1.26–15.4]	0.020
NYHA class (+1)	0.86 [0.46–1.62]	0.64			1.17 [0.35–3.76]	0.80		
Atrial fibrillation	1.18 [0.53–2.59]	0.69			0.84 [0.24–3.00]	0.79		
Hemoglobin (+1 g/dL)	1.12 [0.91–1.43]	0.30			0.90 [0.63–1.33]	0.58		

Abbreviations as in Tables 1 and 2

*ICM* ischemic cardiomyopathy, *NSVT* non-sustained ventricular tachycardia

^a^Adjusted for age and sex

In univariable analysis, indexes predictive of unplanned hospitalizations were precedent NSVT and low LVEF (Table 4, *P* = 0.047 and *P* < 0.0001, respectively). These associations remained significant after adjusting for age and sex (Table 4, *P* = 0.031, and *P* < 0.0001, respectively). Even when the indexes which tended to be associated with unplanned hospitalization such as NYHA class and AF were included in the multivariable model, precedent NSVT and low LVEF remained significant (Table 4).

**Table 4:** Predictors of unplanned hospitalizations due to heart failure

	Univariable analysis		Multivariable analysis		Adjusted model^a^	
	Hazard ratio	*P* value	Hazard ratio	*P* value	Hazard ratio	*P* value
LVEF (+1%)	0.91 [0.87–0.95]	< 0.0001	0.91 [0.86–0.96]	0.0003	0.91 [0.87–0.95]	< 0.0001
NSVT	2.03 [1.01–4.09]	0.047	2.18 [1.08–4.39]	0.031	2.16 [1.07–4.36]	0.031
NYHA class (+1)	1.81 [0.98–3.35]	0.057	0.82 [0.35–1.84]	0.64		
Atrial fibrillation	1.80 [0.90–3.60]	0.099	1.68 [0.82–3.41]	0.15		
Hemoglobin (+ 1 g/dL)	0.87 [0.73–1.06]	0.16				
Ischemic cardiomyopathy	1.43 [0.68–3.03]	0.35				

Abbreviations as in Tables 1 and 2

*ICM* ischemic cardiomyopathy, *NSVT* non-sustained ventricular tachycardia

^a^Adjusted for age and sex

In subgroup analyses, precedent NSVT was an independent predictor of unplanned hospitalizations only in ICM patients (HR: 4.08, 95% CI 1.61–10.3, *P* = 0.0030, Additional file 1: Table S1), while lower LVEF was an independent predictor of hospitalizations in both ICM and NICM patients (*P* = 0.0015, and *P* = 0.0005, respectively, Additional file 1: Table S1).

The primary endpoint was associated with significant coronary stenosis in 4 patients (11%), who underwent PCI (Additional file 2: Figure S1). Nine patients underwent VT ablation. During follow-up after VT ablation, 8 out of these 9 patients were free from further AIT. Of the 23 patients treated with optimal medical therapy alone, 11 (48%) experienced recurrent AIT.

### NSVT burden and duration

During follow-up, the number of precedent NSVT events was significantly higher in patients with AIT as compared to those without AIT (5.6 ± 6.4 vs. 0.5 ± 1.6 times during preceding 6 months, *P* < 0.0001). The maximal duration of preceding NSVT events was also significantly longer in patients with AIT during follow-up (6.4 ± 5.4 vs. 0.7 ± 1.8 s, 18.6 ± 15.6 vs. 2.0 ± 5.3 beats, respectively, *P* < 0.0001). The recorded rate of NSVT did not differ significantly between patients with and without AIT during follow-up (177 ± 20 vs. 180 ± 28 bpm, *P* = 0.61).

Receiver operating characteristic (ROC) analysis revealed that ≥ 1 episode of NSVT within 6 months prior to the index ICD check predicted AIT with 82% specificity and 81% sensitivity during follow-up (C-statistic = 0.84). In regard to the duration of precedent NSVT, ROC analysis demonstrated that a cut-off of 2.3 s or 7 beats of precedent NSVT demonstrated 88% or 86% specificity and 75% or 78% sensitivity for AIT, respectively (C-statistic = 0.86, and 0.86, respectively).

## Discussion

This study demonstrated that in patients who received primary prevention ICDs, those with precedent NSVT detected on ICD interrogation have a higher incidence of subsequent appropriate ICD therapies (shocks and ATP) as compared to the patients without NSVT. Furthermore, ischemic cardiomyopathy patients with NSVT had a higher risk of hospitalization due to worsening heart failure, whereas those with non-ischemic cardiomyopathy and NSVT did not.

Our data demonstrated that asymptomatic NSVT could predict appropriate ICD therapy in both ICM and NICM patients. Previous studies have reported that AIT in itself was associated with a worse prognosis [5, 6]. Our subgroup analysis demonstrated that precedent asymptomatic NSVT predicted unplanned hospitalizations in ICM patients. In ICM patients, the computed time-dependent AUC for unplanned hospitalization was 0.69 (95% CI 0.56–0.82) using only the LVEF as a variable in the model and this was improved to 0.75 (0.63–0.87) by incorporating the documentation of prior NSVT into the model.

Dating back to the pre-ICD era, prior studies have demonstrated the association of NSVT with mortality in some patient cohorts, whereas others reported conflicting results [7–12]. However, multiple trials since have failed to demonstrate the efficacy of anti-arrhythmic drug therapy to suppress ventricular arrhythmias in improving patient outcomes [14–16]. In contrast, anti-arrhythmic drugs, particularly class-Ic drugs, were reported to increase sudden cardiac death. Since the 1990s, advances in cardiovascular therapies such as heart failure medications, ICDs, cardiac resynchronization therapy, and percutaneous coronary intervention resulted in improved survival of these patients [17].

Jiménez-Candil et al. [18] reported that NSVT episodes identified on ICD interrogation in patients with reduced LVEF were an independent predictor of cardiac mortality, hospitalization and AIT. They included both primary- and secondary-prevention ICD patients. For primary-prevention ICD patients, Verma et al. reported using Holter monitoring prior to ICD implantation the presence of NSVT as an independent predictor of AIT [19]. In the MADIT-CRT trial, NSVT were associated with a poorer prognosis and less reverse remodeling of the LV [20]. These data are in line with our findings that NSVT may constitute a therapeutic target in a contemporary heart failure patient cohort with primary prophylactic ICD implantation.

Recently, it has been shown that rapid-rate NSVT was associated with AIT and all-cause mortality [21]. This study did not include NSVT in the monitor zone. In our study, there was no significant difference in the rate of NSVT between patients with and without AIT during follow-up. A possible explanation could be the defined time window of NSVT documentation. Zhou et al. documented NSVT during an on-going study and assessed the correlation between the outcomes. NSVTs in their study may reflect acute worsening of HF. In our study, however, NSVT documentation was assessed for the 6-month period prior to enrollment, in order to assess its utility as a predictor of outcomes.

Our follow-up data (Additional file 2: Figure S1) indicated that ischemia evaluation before antiarrhythmic therapy is an important step. Simultaneously, simple medical intensification after AIT may not be sufficient to prevent further ventricular arrhythmias. Based on these results, early proactive catheter intervention for asymptomatic NSVT may impact on future ICD shock delivery or worsening heart failure, particularly with ischemic cardiomyopathy as the underlying heart disease. The optimal timing of VT ablation has not yet been determined. Prognostic improvement after “prophylactic” VT ablation has not been definitively demonstrated in previous trials, in which the patients were recruited after experiencing at least one sustained VT event [22, 23]. Our data suggest the hypothesis that prophylactic VT ablation in ICD patients with NSVT may improve a patient’s prognosis. This hypothesis is currently being tested in an ongoing prospective randomized clinical trial (ClinicalTrials.gov, NCT03147027).

The present study has some limitations. This study is a prospective, observational single-centre study. These results should be further confirmed by larger multi-centre studies. Furthermore, with larger numbers of patients with NICM, the risk of worsening HF according to the precedent NSVT should be also reevaluated. Second, our ICD programming is based on our institutional standards, which did not include a single high rate therapy zone with prolonged detection. However, our VF zone setting is similar to the MADIT-RIT high-rate group, although we set the cutoff rates 10 bpm higher than the MADIT-RIT high-rate group. Our VT zone had prolonged detections, and we set an additional monitor zone. Third, the occurrence of AIT in our cohort was 23% during approximately 3 years of follow-up. This is a high incidence and may reflect more unwell population in our cohort. The algorithms for NSVT documentation are different according to the different device manufacturers, with the shortest NSVT detection at 5–6 beats. Therefore, it is uncertain if shorter episodes of NSVT such as 3–4 beats may also confer an increased risk of subsequent AIT. Finally, the IEGM morphological characteristics of NSVT were not assessed in this study. This clinical significance should be further investigated in future studies.

## Conclusions

Precedent NSVT in ischemic- and non-ischemic cardiomyopathy patients who received primary prevention ICD implantation predicted subsequent appropriate ICD therapies. In patients with ICM, precedent NSVT was an independent predictor of unplanned hospitalization due to worsening heart failure. Based on these results, the efficacy of specific interventions targeting NSVT in this patient population should be investigated in future studies.

## Supplementary information

**Additional file 1: Table S1.** Predictors of unplanned hospitalizations due to heart failure according to etiologies of cardiomyopathies.

**Additional file 2: Figure S1.** Clinical course after appropriate ICD therapy. The clinical courses after appropriate ICD therapy (AIT) are described. In total, 25 patients underwent coronary angiography or scintigraphy to exclude coronary stenosis and 4 patients underwent further percutaneous coronary intervention due to significant coronary stenosis (> 90%). Recurrent VT with AIT was noted in 11 patients out of 23 patients with medical therapy intensification, and in 1 patient out of 9 patients with VT ablation (see text in detail).

## Data Availability

The datasets used or analysed during the current study are available from the corresponding author on reasonable request.
