# Supplementary material for: Clinical significance of precedent asymptomatic non-sustained ventricular tachycardias on subsequent ICD interventions and heart failure hospitalization in primary prevention ICD patients
Source: Eur J Med Res. 2020 Mar 17;25:5. doi: 10.1186/s40001-020-0401-x (PMC7076933; doi:10.1186/s40001-020-0401-x)
Supplement: Supplementary file 1 — Additional file 1: Table S1. Predictors of unplanned hospitalizations due to heart failure according to etiologies of cardiomyopathies. [file 40001_2020_401_MOESM1_ESM.docx]

**Table S1. Predictors of unplanned hospitalizations due to heart failure according to etiologies of cardiomyopathies**

|  | **Ischemic Cardiomyopathy** | | | | **Non-ischemic Cardiomyopathy** | | | |
| --- | --- | --- | --- | --- | --- | --- | --- | --- |
|  | **Univariable analysis** | | **Adjusted model*** | | **Univariable analysis** | | **Adjusted model*** | |
|  | **Hazard Ratio** | **P value** | **Hazard Ratio** | **P value** | **Hazard Ratio** | **P value** | **Hazard Ratio** | **P value** |
| LVEF (+1%) | 0.93 [0.88-0.97] | 0.0033 | 0.92 [0.87-0.96] | 0.0015 | 0.85 [0.77-0.93] | 0.0022 | 0.85 [0.77-0.93] | 0.0005 |
| NSVT | 2.95 [1.23-7.05] | 0.015 | 4.08 [1.61-10.3] | 0.0030 | 0.78 [0.16-3.69] | 0.75 | 0.66 [0.13-3.25] | 0.60 |
| NYHA Class (+1) | 1.86 [0.90-3.85] | 0.093 |  |  | 1.62 [0.55-4.84] | 0.38 |  |  |
| Atrial fibrillation | 1.86 [0.81-4.30] | 0.14 |  |  | 1.79 [0.50-6.43] | 0.37 |  |  |
| Hemoglobin (+1 g/dL) | 0.96 [0.77-1.23] | 0.73 |  |  | 0.72 [0.51-1.02] | 0.058 |  |  |
