# Supplementary figures and images for: Clinical significance of precedent asymptomatic non-sustained ventricular tachycardias on subsequent ICD interventions and heart failure hospitalization in primary prevention ICD patients
Source: Eur J Med Res. 2020 Mar 17;25:5. doi: 10.1186/s40001-020-0401-x (PMC7076933; doi:10.1186/s40001-020-0401-x)

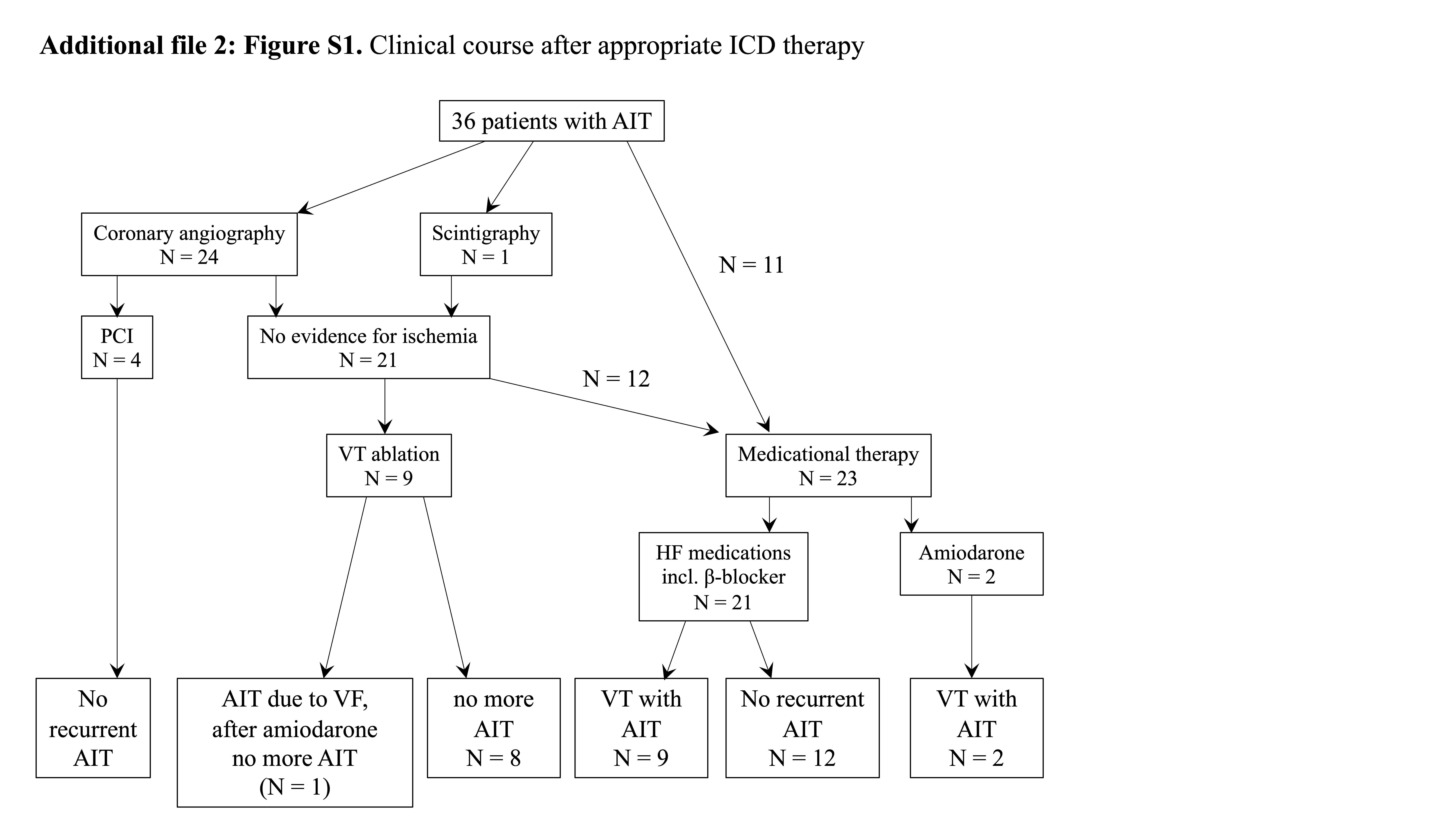

Supplement: Supplementary file 2 — Additional file 2: Figure S1. Clinical course after appropriate ICD therapy. The clinical courses after appropriate ICD therapy (AIT) are described. In total, 25 patients underwent coronary angiography or scintigraphy to exclude coronary stenosis and 4 patients underwent further percutaneous coronary intervention due to significant coronary stenosis (> 90%). Recurrent VT with AIT was noted in 11 patients out of 23 patients with medical therapy intensification, and in 1 patient out of 9 patients with VT ablation (see text in detail). [file 40001_2020_401_MOESM2_ESM.tiff]
